# Primary Health Care Facility Preparedness for Outpatient Service Provision During the COVID-19 Pandemic in India: Cross-Sectional Study

**DOI:** 10.2196/19927

**Published:** 2020-06-01

**Authors:** Suneela Garg, Saurav Basu, Ruchir Rustagi, Amod Borle

**Affiliations:** 1 Department of Community Medicine Maulana Azad Medical College New Delhi India

**Keywords:** primary health care, COVID-19, pandemic, health systems, India

## Abstract

**Background:**

Primary health centers (PHCs) represent the first tier of the Indian health care system, providing a range of essential outpatient services to people living in the rural, suburban, and hard-to-reach areas. Diversion of health care resources for containing the coronavirus disease (COVID-19) pandemic has significantly undermined the accessibility and availability of essential health services. Under these circumstances, the preparedness of PHCs in providing safe patient-centered care and meeting the current health needs of the population while preventing further transmission of the severe acute respiratory syndrome coronavirus 2 infection is crucial.

**Objective:**

The aim of this study was to determine the primary health care facility preparedness toward the provision of safe outpatient services during the COVID-19 pandemic in India.

**Methods:**

We conducted a cross-sectional study among supervisors and managers of primary health care facilities attached to medical colleges and institutions in India. A list of 60 faculties involved in the management and supervision of PHCs affiliated with the community medicine departments of medical colleges and institutes across India was compiled from an accessible private organization member database. We collected the data through a rapid survey from April 24 to 30, 2020, using a Google Forms online digital questionnaire that evaluated preparedness parameters based on self-assessment by the participants. The preparedness domains assessed were infrastructure availability, health worker safety, and patient care.

**Results:**

A total of 51 faculties responded to the survey. Each medical college and institution had on average a total of 2.94 (SD 1.7) PHCs under its jurisdiction. Infrastructural and infection control deficits at the PHC were reported in terms of limited physical space and queuing capacity, lack of separate entry and exit gates (n=25, 49%), inadequate ventilation (n=29, 57%), and negligible airborne infection control measures (n=38, 75.5%). N95 masks were available at 26 (50.9%) sites. Infection prevention and control measures were also suboptimal with inadequate facilities for handwashing and hand hygiene reported in 23.5% (n=12) and 27.4% (n=14) of sites, respectively. The operation of outpatient services, particularly related to maternal and child health, was significantly disrupted (*P*<.001) during the COVID-19 pandemic.

**Conclusions:**

Existing PHC facilities in India providing outpatient services are constrained in their functioning during the COVID-19 pandemic due to weak infrastructure contributing to suboptimal patient safety and infection control measures. Furthermore, there is a need for effective planning, communication, and coordination between the centralized health policy makers and health managers working at primary health care facilities to ensure overall preparedness during public health emergencies.

## Introduction

The coronavirus disease (COVID-19), which is caused by severe acute respiratory syndrome coronavirus 2 (SARS-CoV-2), has resulted in an unprecedented global health crisis [1]. The COVID-19 pandemic in India has already recorded more than 118,446 cases and 3583 deaths (as of May 23, 2020), with cases reported in every state of the country [2].

Healthy systems in lower and lower-middle income countries are experiencing major challenges in coping with the COVID-19 pandemic due to the high pre-existing vulnerability from the limited public health infrastructure combined with the diversion of essential medical resources for the provision of dedicated care and management to presumptive COVID-19 cases [3-5]. In India, the second-most populous country in the world, several secondary and tertiary care hospitals that cater to millions of daily outpatients have been converted into temporary dedicated COVID-19 hospitals to provide care to the patients with moderate and severe COVID-19 as per the designated clinical criteria [6,7]. Consequently, the health care needs of patients with chronic diseases and maternal and child health requires alternative primary care service delivery. The World Health Organization (WHO) has also warned of the increased likelihood of resurgence in outbreaks of vaccine-preventable diseases due to the subversion of routine immunization services during the COVID-19 pandemic [8]. In addition, pandemic preparedness at primary care facilities that enable the early management of novel infectious diseases in communities with the reduction in their potential morbidity and mortality is well-established [9,10]. In India, a network of over 25,000 primary health centers (PHCs), the first and lowest health tier, provide essential preventive, promotive, and curative health services such as maternal and child health, essential drugs, and health education in the rural, suburban, and underserved hard-to-reach areas [11]. Furthermore, all medical colleges and institutions in India, as per the mandate of the reorientation of medical education scheme, are linked to primary health facilities in rural and urban areas through the community medicine department [12]. Hence, the preparedness of PHCs in providing safe patient-centered care for meeting the current health needs of the population and preventing further transmission of the SARS-CoV-2 infection is crucial.

The study objective was to determine the primary health care facility preparedness toward the provision of safe outpatient services during the COVID-19 pandemic in India

## Methods

### Design and Settings

We conducted a cross-sectional study among supervisors and managers of primary health care facilities attached to medical colleges and institutions anywhere in India, either in the government or private setting. We collected the data for 7 days from April 24 to 30, 2020, through means of a web-based survey for self-assessment by PHC managers and supervisors. The preparedness domains assessed were infrastructure availability, health worker safety, and patient care [13].

### Procedure

We collected data through a rapid survey using a pretested Google Forms online digital questionnaire (Multimedia Appendix 1). A nonrandom convenient sampling method was used to select the study participants. A list of 60 faculty involved in the management and supervision of PHCs affiliated to the community medicine departments of medical colleges and institutes across India was compiled from an accessible private organization member database. These members were sent an invitation to participate in the study through email and instant messages containing the Google Form survey link. Since the sampling unit was a medical college or institution, we selected only one potential respondent from each site to prevent duplication.

### Operational Definitions

Operations (functionality) of any specific health service through outpatient clinics conducted at the PHCs was assessed in terms of continuity of service provision to patients and beneficiaries at the time of the survey. Pre-COVID-2019 refers to the period of service delivery at the PHCs until February 2020. Post-COVID-2019 refers to the period of PHC service delivery at the time of the survey. Adequate ventilation refers to the availability of cross-ventilation with separate doors and windows at the site of service provision to the patients. Adequate handwashing facilities refers to the availability of running water and soap for patients. Adequate hand hygiene facilities refers to the availability of alcohol-based hand sanitizer for health staff.

### Statistical Analysis

The Google Form data was exported into Microsoft Excel 2013 (Microsoft Corporation) software and cleaned and analyzed using SPSS version 25 (IBM Corp). Results were expressed in frequency, proportions, and 95% confidence intervals for categorical variables, and mean for continuous variables. The significance of the difference between proportions was assessed using the chi-square test. A *P*<.05 was considered statistically significant.

### Ethical Considerations

The study was approved and exempted from full review by the Institutional Ethics Committee (F.1/IEC/MAMC/(73/01/ 2020/No68). The consent of the participants was implied as participation in the study was voluntary.

## Results

A total of 51 faculty from various medical institutions and colleges across India responded to the survey for a net response rate of 85% (n=51/60). The participants were from Northern India (n=31, 60.7%), Southern India (n=6, 11.7%), Western India (n=7, 13.7%), and Eastern and Northeastern India (n=7, 13.7%).

Infrastructure preparedness of the primary health facilities was assessed in terms of the total number of rooms that were available for service provision. Each medical college or institution had on average a total of 2.94 (SD 1.7) PHCs under its jurisdiction. The median number of both urban and rural PHCs attached to each of the medical colleges and institutions was 1. The average number of rooms in the largest and the smallest PHC for patient-care purposes was 3.37 and 1.77, respectively. Furthermore, each PHC was equipped on average with 2.71 rooms for the provision of various health care services.

Patient care and service provision preparedness were evaluated by comparing the outpatient department (OPD) clinic functionality and the number of patients and beneficiary services. Before the onset of the COVID-19 epidemic in India, the participant colleges and institutions were providing on average antenatal care (ANC) and immunization services at their sites to 26.5 and 41.4 clients, respectively. However, outpatient services were significantly disrupted during the COVID-19 epidemic. Among the OPD clinics at the PHC sites, the maximum reduction in clinic operations was reported for the noncommunicable diseases (NCD) and the immunization clinics; ANC services were less disrupted. In contrast, the general OPDs were least disrupted (Table 1). Furthermore, fever (flu) clinics had been started at 72.4% (n=37) of the sites to screen patients reporting with symptoms of influenza-like illnesses (fever, dry cough, or respiratory difficulties) for suspected COVID-19 and initiate appropriate referral services when necessary. On average, 30 patients attended these fever clinics each day in the sites where such dedicated clinics were available.

**Table 1:** Comparison of outpatient services (N=51) functionality during the COVID-19 epidemic in India during the study period (April 24-30, 2020).

OPD^a^ facility	Pre-COVID-19^b^		Post-COVID-19		*P* value	
	n (%)	95% CI	n (%)	95% CI		
ANC^c^	48 (94.1)	83.8-98.8	33 (67.3)	50.1-77.6		<.001
Immunization	48 (94.1)	83.8-98.8	26 (53.1)	36.6-65.2		<.001
Animal bite	23 (45.1)	31.1-59.7	14 (27.4)	15.9-41.7		.06
NCD^d^	47 (92.1)	81.1-97.8	21 (41.1)	27.6-55.8		<.001
General	51 (100.0)	0.93-100	42 (82.3)	36.5-46.0		.002

^a^OPD: outpatient department.

^b^COVID-19: coronavirus disease.

^c^ANC: antenatal care.

^d^NCD: noncommunicable diseases.

Safety and infection control in the PHCs was assessed in terms of space for patient queuing, availability of cross ventilation through separate doors and windows, type of entries and exits, and the existing protocol for conducting disinfection measures. Each site, on average, reported a patient queuing capacity of 14.1 persons subject to maintaining minimum physical distancing requirements to reduce the chances of SARS-CoV-2 transmission. Moreover, nearly half (n=25, 49%) of the sites were missing separate or multiple entries and exits (n=27, 52.9%). A majority (n=29, 57%) of the participants reported inadequate ventilation at their PHC sites. Airborne infection control measures were reported as absent in 75.5% (n=38) of sites. Nevertheless, chemical disinfection of the PHCs was being undertaken at most (n=42, 82.4%) sites with daily, alternate day, and less frequent disinfection reportedly conducted in 52.9% (n=27), 13.7% (n=7), and 19.6% (n=10) of the sites, respectively. However, adequate handwashing services for patients were unavailable at 12 (23.5%) sites.

A majority of respondents (n=34, 66.7%) supervising their respective PHC sites lacked adequate confidence in achieving effective segregation of patients with presumptive COVID-19 from other routine beneficiaries for preventing nosocomial transmission of the SARS-CoV-2 infection at their sites. For this reason, a majority (n=30, 58.8%) of the participants were disinclined toward operating dedicated fever clinics simultaneously with any special OPD clinics.

The safety of health workers was evaluated in terms of the provision of personal protective equipment (PPE) to the health staff and access to hand hygiene facilities. PPE suits were available at 14 (27.4%) sites, N95 masks at 26 (50.9%) sites, and only surgical masks were available at 19 (39.3%) sites. Hand hygiene facilities for PHCs were considered inadequate at 14 (27.4%) sites. Training related to the safe and effective management of patients with presumptive COVID-19 had been previously provided at 40 (78.4%) of the sites to their health staff.

## Discussion

### Principal Findings

The maintenance of essential care health services on an outpatient basis during the aftermath of the COVID-19 pandemic is a major public health challenge. Our study findings indicate that the provision of essential outpatient health care service were disrupted in a significant proportion of PHCs across India with the onset of the COVID-19 pandemic and the rapid escalation of cases. Furthermore, suboptimal infrastructural capacity at most PHCs, poor ventilation, negligible airborne infection control measures, and constraints in achieving minimum physical distancing requirements among patients needed to reduce the risk of COVID-19 transmission [14] possibly precluded the expansion of screening and referral of presumptive COVID-19 cases at these sites.

The public health measures undertaken for containing the COVID-19 epidemic in India significantly contributed to the decline in the provision of essential services at the PHCs. First, India enforced a strict nationwide lockdown from March 23, 2020, onwards (which is continuing at the time of writing), including ceasing all public transport, resulting in diminished health care accessibility [15]. Second, there was a diversion of health care staff, especially doctors and nurses, for COVID-19–related duties. Moreover, even frontline community health workers, including the accredited social health activists, were engaged in the surveillance and contact tracing activities related to COVID-19 [16]. This resulted in the absence of community mobilization of women and caregivers for continuing with immunization and regular ANC services at the PHCs. Third, the feasibility of separating waiting areas for immunization services from curative services and adherence to physical distancing at the health facilities as recommended by the WHO [17] was not possible at several sites due to infrastructural limitations. Fourth, parents of immunization eligible children and antenatal women possibly refrained from visiting primary health facilities due to increased risk perception of contracting the coronavirus infection from other patients.

Primary care providers are known to be at increased risk of getting infected with new infectious diseases, especially when handling patients with acute respiratory illnesses during epidemics [18,19]. The inadequate availability of PPE for health care providers during the COVID-19 pandemic has also been observed worldwide [20]. Under these circumstances, the allocation of PPE has been subject to expert criteria that recommend limiting the provision of N95 masks to only those health care providers who are directly involved in the management of confirmed COVID-19 cases [21]. Consequently, primary care providers in resource-constrained settings, working in enclosed small clinic spaces that lack adequate ventilation and are likely overcrowded, are rendered highly vulnerable to COVID-19 in the absence of effective PPE provision. In our analysis, we found that only 1 in 2 medical colleges and institutions were able to provide N95 masks to the health care providers at their primary health care facilities. However, the adequacy of the supply of N95 masks was not assessed in this study.

According to the report of the National Health Policy (2017), only 1 in 5 Indians in rural India use outpatient services at public (government) health facilities due to perceived deficiencies in standards of care arising from dilapidated infrastructure and nonavailability of essential services and drugs [22]. Considering the deficiencies in physical infrastructure at PHC sites combined with the ubiquitous availability of cheap mobile telecommunication services, the potential role of telemedicine services to maintain continuity of care should be considered in such settings [23].

Finally, the functionality of fever or flu clinics is considered a crucial primary care role in the preliminary assessment, counseling and reassurance, and referral of patients reporting with influenza-like illness during pandemics similar to COVID-19. However, in India, government guidelines have stipulated the functioning of fever clinics at primary care facilities subject to the availability of adequate space [7]. Nevertheless, in this study, we found nearly 3 in 4 institutions were operating fever clinics at their PHC facilities despite the obvious infrastructural limitations.

### Study Limitations

First, due to the convenient sample, representativeness was limited as a majority of sites were restricted to Northern India. Second, the survey was based on self-assessment, which can be subject to bias. Third, we conducted a cross-sectional survey and did not assess prospective change in the OPD clinic operations and service provisions during the COVID-19 epidemic in India. Fourth, we did not consider factors related to facility preparedness like health education and the monitoring and surveillance of health events due to the absence of objective evaluation through an external observer. Fifth, reasons for nonfunctionality of the OPD clinics, which were either due to logistical constraints or because of nonreporting by patients and beneficiaries were not ascertained in this survey.

### Conclusion

Existing PHC facilities in India providing outpatient care during the COVID-19 epidemic are constrained in their functioning by weak infrastructure contributing to suboptimal patient safety and infection control measures. Most PHCs reduced essential OPD services and instead were running dedicated clinics for screening and referral of patients with suspected COVID-19 reporting with symptoms of influenza-like illnesses. However, health care managers must address the risks of nosocomial infection in these settings when operationalizing simultaneous COVID-19 screening and special OPD clinics for NCDs, ANC, and care for children younger than 5 years that cater to populations highly susceptible to COVID-19 [24].

### Recommendations

The COVID-19 pandemic is still ensuing, and there are projected second waves and considerable time until development of any effective vaccine, generation of herd immunity, and ultimate pandemic resolution [25]. Findings from this study emphasize the need for effective planning, communication, and coordination between the centralized health policy makers and health managers working at primary care settings to ensure overall preparedness. For instance, the government of India, after 3 weeks of lockdown (April 14, 2020), issued a general guideline to the states to make best efforts toward continuing with essential health services at peripheral health facilities as per their feasibility [26]. Subsequently, on May 20, 2020, the government of India issued a comprehensive guidance note that re-emphasized the need for continuing both facility and outreach immunization services except in the containment zones (areas having a relatively higher number of COVID-19 cases and shorter time to doubling of cases) [27,28]. These lessons are suggestive of the need for maintaining effective channels of health communication between various stakeholders for ensuring continuity of essential services during any future waves of the COVID-19 pandemic.

In the medium- and long-term, governments both at the state and center should considerably invest in infrastructure, capacity building, and the strengthening of primary health care services to ensure their effective functioning during public health emergencies [29]. Under the Ayushman Bharat National Health Protection, the government of India has envisaged the upgrading and developing of 150,000 primary care facilities throughout India [30]. Nevertheless, to strengthen PHC in India and significantly augment their capacities and roles during pandemics, an emphatic focus on infrastructure development, especially in terms of spacing, ventilation, and infection control, warrants high prioritization.

## Appendix

Multimedia Appendix 1 Survey questionnaire.

## Abbreviations

ANC: antenatal care
COVID-19: coronavirus disease
PHC: primary health center
NCD: noncommunicable diseases
OPD: outpatient department
PPE: personal protective equipment
SARS-CoV-2: severe acute respiratory syndrome coronavirus 2
WHO: World Health Organization
