# Supplementary material for: Primary Health Care Facility Preparedness for Outpatient Service Provision During the COVID-19 Pandemic in India: Cross-Sectional Study
Source: JMIR Public Health Surveill. 2020 Jun 1;6(2):e19927. doi: 10.2196/19927 (PMC7265797; doi:10.2196/19927)
Supplement: Multimedia Appendix 1 [file publichealth_v6i2e19927_app1.pdf]

ANNEXURE 1. Survey Instrument

Name of the college / institution	
State/UT (where you are working)	1. Assam<br>2. Arunachal Pradesh<br>3. Andhra (Seemandhra)<br>4. Andaman<br>5. Chandigarh<br>6. Chhatisgarh<br>7. Bihar<br>8. Dadra and Nagar Havel<br>9. Delhi<br>10. Goa<br>11. Gujarat<br>12. Haryana<br>13. Himachal Pradesh<br>14. Jharkhand<br>15. Jammu and Kashmir<br>16 Karnataka.<br>17. Kerala<br>18. Maharashtra<br>19.Madhya Pradesh<br>20. Manipur<br>20.. Meghalaya<br>21. Mizoram<br>22. Nagaland<br>23. Odisha<br>24. Punjab<br>25. Rajasthan<br>26. Sikkim<br>27. Uttar Pradesh<br>28. Uttarakhand<br>29. Tamil Nadu<br>30. Telangana<br>31. Tripura<br>32. West Bengal
Organization Type	1. Government<br>2. Private<br>3. PPP/NGO
Total number of primary care facilities where you work or under your jurisdiction	1. One<br>2. Two<br>3. Three<br>4. Four<br>5. Other (Specify)
Total urban primary care facilities where you work or under your jurisdiction	1. One<br>2. Two<br>3. Three<br>4. Others (specify)

Total rural primary care facilities where you work or under your jurisdiction	1. One<br>2. Two<br>3. Three<br>4. Others (specify)
Pre-COVID-19 (Until February 2020) services that were being provided at your primary care facilities<br>(check all that apply)	1. Antenatal care<br>2. Immunization of children and mothers<br>3. NCD/Diabetes clinic<br>4. General OPD
Currently, enlist the services that are being currently provided at your primary care facilities<br>(check all that apply)	1. Fever Clinic<br>2. Antenatal care<br>3. Immunization of children and mothers<br>4. NCD/Diabetes clinic<br>5. General OPD
In the largest of your primary care facilities, how many rooms are available for patient care purposes	1. One<br>2. Two.<br>3. Three<br>4. Four or more
In the smallest of your primary care facilities, how many rooms are available for patient care purposes	1. One<br>2. Two.<br>3. Three<br>4. Four or more
On average, how many rooms are available at your primary care facilities for patient care purposes	1. One<br>2. Two.<br>3. Three<br>4. Four or more
Pre-COVID-19 (Until February 2020), on average, how many ANC clients were provided services at all your primary care health facilities during a single session.	________
Pre-COVID-19 (Until February 2020), on average, how many children were immunized per session at all your primary care health facilities during a single session	_________
What is the total queueing capacity (number of patients that can be queued and seated) after accounting for minimum 1 meter physical distancing requirements in all your health facilities	_________
How many of your primary care facilities have separate entry and exit points	_______________________
How many of your primary care facilities have multiple entry and exit points	_________________________
How confident are you that segregation of fever / presumptive COVID-19 cases from general OPD cases to prevent nosocomial	1 2 3 4 5

coronavirus infection can be maintained in your primary care health facilities<br>(1: Least confidence, 5: Complete confidence)	
Is it feasible to run dedicated fever clinics and special clinics at your primary care facilities (ANC/Immunization/NCD/General)	1. Yes<br>2. No<br>3. Not sure
Do you have adequate handwashing facilities for patients at your primary care facilities?	1. Yes<br>2. No
Do you have adequate masks for patients who may request so?	1. Yes<br>2. No
Do you have adequate ventilation at your primary care facilities? (cross-ventilation, with separate doors and windows)	1. Yes<br>2. No<br>3. Not sure
Are there any specific airborne infection control measures operational in your primary care facilities?	1. Yes<br>2. No
